# Effect of Non-surgical Periodontal Therapy on Levels of Ghrelin and Tumor Necrosis Factor-Alpha (TNF-α) in the Gingival Crevicular Fluid of Healthy and Periodontitis Patients With and Without Type 2 Diabetes Mellitus: A Clinico-Biochemical Study

**DOI:** 10.7759/cureus.95240

**Published:** 2025-10-23

**Authors:** M B Kaustubh Ram, Savitha A N, B S Jagadish Pai, Praveen Jayaram

**Affiliations:** 1 Periodontology, The Oxford Dental College, Bengaluru, IND

**Keywords:** ghrelin, nspt, periodontitis, t2dm, tnf-α

## Abstract

Purpose: Periodontitis, a chronic inflammatory disease, is closely associated with type 2 diabetes mellitus (T2DM). Ghrelin, a stomach-derived peptide hormone, may influence periodontal health by modulating inflammation through its receptor growth hormone secretagogue receptor (GHS-R1a), notably by inhibiting lipopolysaccharide (LPS)-induced tumor necrosis factor-alpha (TNF-α) production in oral epithelial cells.

Aim: To compare and correlate the gingival crevicular fluid (GCF) ghrelin and TNF-α levels in healthy individuals and stage III grade B periodontitis patients with and without T2DM before and three months after non-surgical periodontal therapy (NSPT).

Materials and methods: Ninety subjects were segregated into three groups: Group I (healthy), Group II (Stage III Grade B periodontitis), and Group III (periodontitis with controlled T2DM). Clinical parameters such as plaque index (PI), gingival index (GI), bleeding on probing (BoP), probing pocket depth (PPD), and interdental clinical attachment loss (ICAL) were recorded along with GCF samples which were collected and analysed at baseline and at the end of three months post NSPT.

Results: Baseline GCF levels of ghrelin and TNF-α differed significantly among the three groups. Three months after NSPT, all the groups exhibited a significant reduction in ghrelin and TNF-α levels in GCF, with the greatest reduction observed in Group III.

Conclusion: Our findings suggest that ghrelin and TNF-α levels may serve as useful biomarkers for evaluating periodontal disease severity and treatment response, especially in patients with T2DM. The study also confirms the effectiveness of NSPT in reducing inflammation, highlighting its critical role in managing periodontitis among individuals with systemic conditions like T2DM.

## Introduction

Periodontitis is a persistent and complex inflammatory condition primarily influenced by the buildup of dental plaque which leads to gradual deterioration of structures supporting the teeth, such as the periodontal ligament and alveolar bone. Research has shown that numerous systemic diseases like type 2 diabetes mellitus (T2DM) are associated with periodontitis [1].

T2DM stands as a chronic metabolic condition marked by elevated blood glucose levels due to either insulin resistance or inadequate insulin production [2]. T2DM is linked with various complications, among which periodontitis is notable. The correlation between T2DM and periodontitis is noteworthy, as individuals with T2DM face an increased likelihood of developing periodontitis [3]. This underscores the importance of focusing on periodontal health in T2DM management to improve glycaemic control and reduce complications.

Gingival crevicular fluid (GCF) is a physiologic fluid secreted in the gingival crevice that is classified as an inflammatory exudate during disease or serum transudate during health. It plays a crucial role in diagnosing, monitoring and evaluating the progression of periodontal disease. It serves as a valuable window for non-invasive analysis of periodontitis by assessing various indicators and biomarkers [4]. Two significant markers of interest considered in this study are ghrelin and tumor necrosis factor-alpha (TNF-α).

Ghrelin, a peptide hormone primarily produced by the stomach, is also found in smaller quantities in various other organs, cells, and tissues including the pituitary gland, salivary glands, teeth, heart, immune system cells, and osteoblasts. Within tissues and circulating in the blood, ghrelin exists in two main forms: des-acylated ghrelin and acylated ghrelin [5].

Both forms of ghrelin play essential roles in physiological processes such as growth hormone secretion, regulation of food intake, and energy metabolism. However, acylated ghrelin significantly impacts periodontal health by modulating the immune system, interacting with inflammatory cytokines, and potentially influencing bone metabolism [6].

Inflated ghrelin levels have been noted in chronic inflammatory conditions such as periodontitis [6]. Conversely, ghrelin levels exhibit a drop in T2DM and associated metabolic syndromes as well [7]. Ghrelin has been found to modulate the output of pro-inflammatory cytokines, such as interleukin (IL) 1β and TNF-α, induced by lipopolysaccharide (LPS), showcasing its potent immunoregulatory properties [8].

TNF-α is a cogent inflammatory cytokine primarily secreted by macrophages and monocytes during acute inflammatory responses [9]. Its presence is not limited to systemic circulation; it can also be found in saliva and GCF in both healthy individuals and those with periodontitis [10].

In periodontitis, elevated levels of TNF-α in saliva and GCF closely correlate with the extent of tissue damage and the degree of host response. This suggests a significant involvement of TNF-α in the pathogenesis of periodontal disease, contributing to tissue destruction and exacerbating the inflammatory process [10].

Studies suggest that TNF-α levels are linked to insulin resistance and beta cell function in individuals with T2DM. TNF-α is implicated in both the initiation and progression of T2DM [11], as evidenced by its heightened presence in T2DM patients against those without the condition, highlighting its role in the emergence of disease.

To the best of our knowledge, this is the first study that has correlated ghrelin, TNF-α and periodontal disease before and after non-surgical periodontal therapy (NSPT). Thus, our study aims to explore the relationship between ghrelin and TNF-α in periodontal wellness and disease in the GCF of T2DM patients.

## Materials and methods

Ethical permission for the study was obtained from the Institutional Ethical Committee and Review Board, The Oxford Dental College (TODC/018/ECAL/2022-23). The research was conducted in accordance with the principles outlined in the Declaration of Helsinki (1975), including its most recent revision in 2013. 

The study included a total of 90 participants, who were equally distributed into three groups after evaluation based on the inclusion and exclusion criteria (Table 1). Group I consisted of systemically and periodontally healthy individuals. Group II comprised subjects diagnosed with generalized Stage III Grade B periodontitis without T2DM, characterized by probing depth (PD) ≥ 6 mm, clinical attachment loss (CAL) ≥ 5 mm, and hemoglobin A1c (HbA1c) ≤ 5.7%. Group III included subjects with generalized Stage III Grade B periodontitis and T2DM, defined by PD ≥ 6 mm, CAL ≥ 5 mm, and HbA1c < 7%.

**Table 1:** Inclusion and Exclusion criteria HbA1c: Hemoglobin A1c, T2DM: type 2 diabetes mellitus

INCLUSION CRITERIA
Subjects of either sex, between the ages of 20 and 60 years.
Presence of at least 20 natural teeth.
Patients with diagnosis of generalised stage III grade B periodontitis. (Periodontal health, gingivitis and periodontitis definition will be based on the classification of periodontitis and peri-implant diseases and conditions as proposed in 2017 World Workshop) [12].
Subjects with T2DM (HbA1c < 7%)
Subjects without T2DM (HbA1c < 5.7%)
EXCLUSION CRITERIA
Obese individuals, BMI >25 kg/m^2^, waist circumference of ≥ 90 cm for men and ≥ 80cm for women according to Asian Pacific Perspective Obesity-WHO 2000 [13].
Individuals who have received periodontal therapy in the last six months.
Individuals with systemic diseases other than type 2 diabetes mellitus (HbA1c ≥ 7.0%)
Individuals who have history of antimicrobial and anti-inflammatory therapy in last six months.
Smokers
Pregnant/lactating and postmenopausal women.

**Figure 1:**
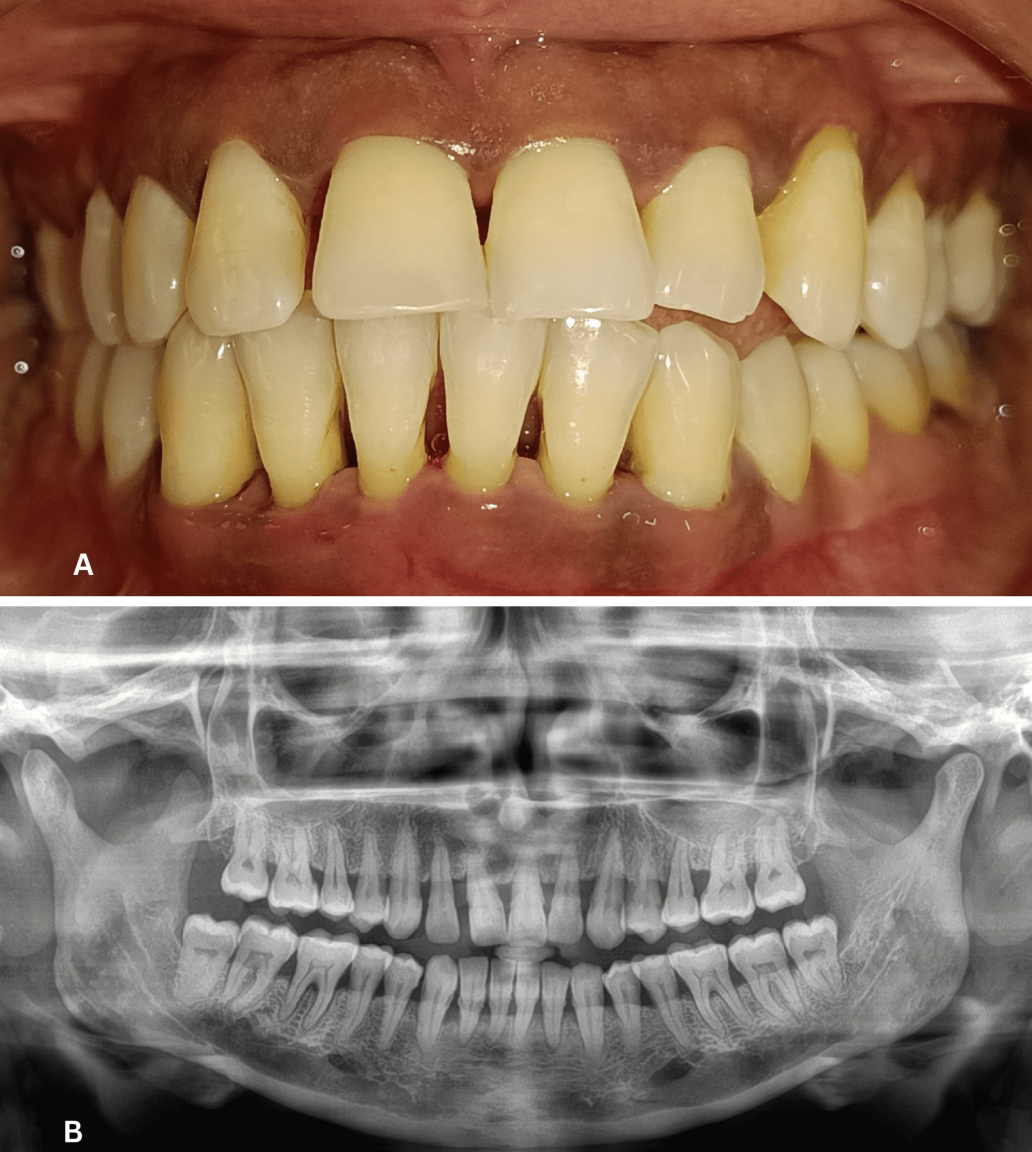
(A) Clinical photograph of Group II (Periodontitis) and (B) its corresponding radiograph.

Participant recruitment and selection were performed through a comprehensive evaluation process, which included recording of case history, medical and dental history, chief complaints, clinical examination, and radiographic assessment. Baseline clinical parameters - plaque index (PI), gingival index (GI), bleeding on probing (BOP), PD, and interdental clinical attachment loss (ICAL) - were recorded one day prior to GCF collection to avoid sample contamination. Radiographs were used to confirm site-specific periodontal involvement (Figure 1).

**Figure 2:**
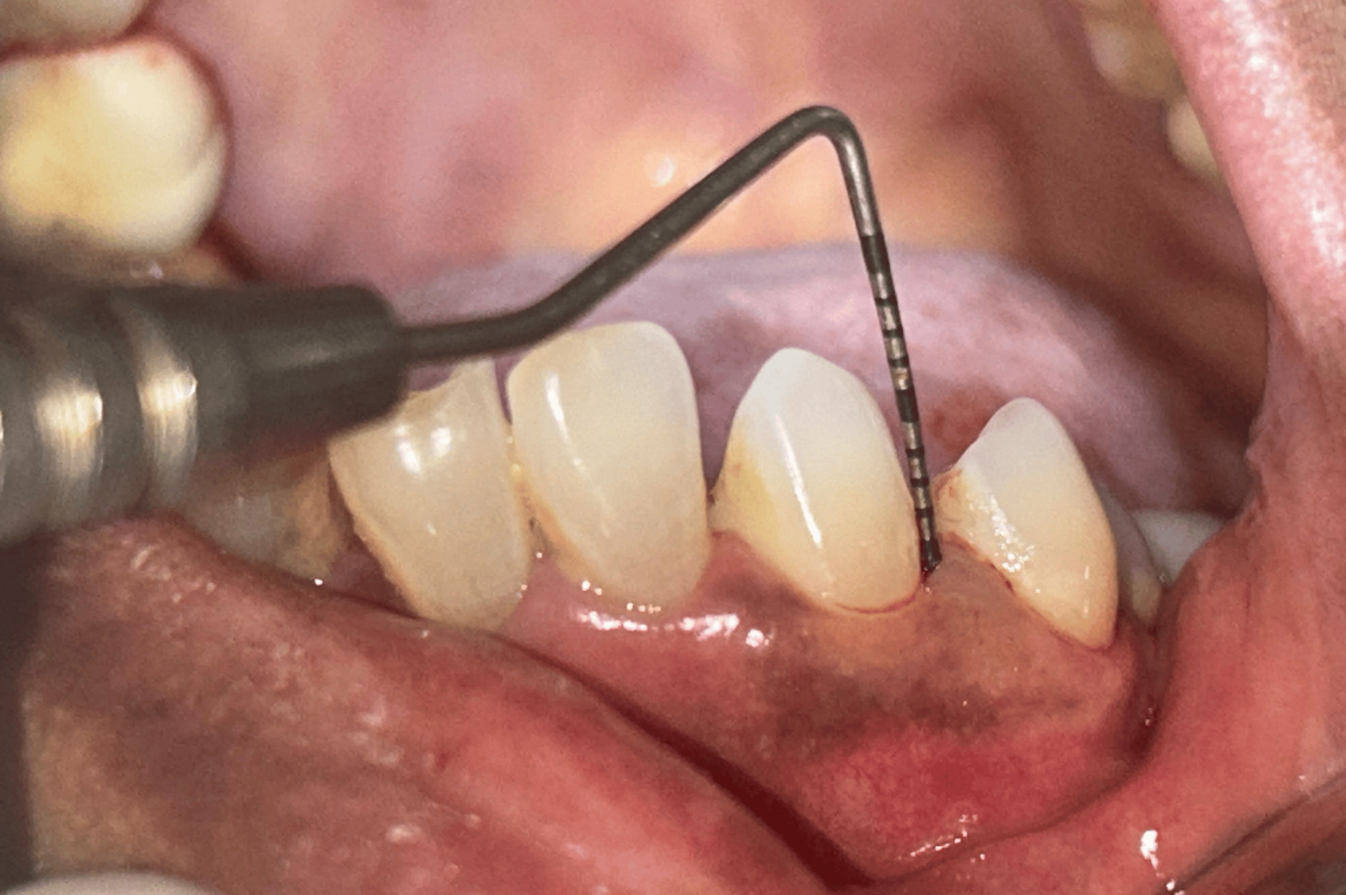
Measuring probing depth using UNC-15 probe

All measurements were performed by a standardized examiner using a UNC-15 periodontal probe to ensure reproducibility and accuracy (Figure 2).

Site selection was clearly defined: healthy sites were considered as those with BOP < 10% and PD ≤ 3 mm, whereas periodontitis sites were defined as those with BOP > 10%, PD ≥ 6 mm, and ICAL ≥ 5 mm.

**Figure 3:**
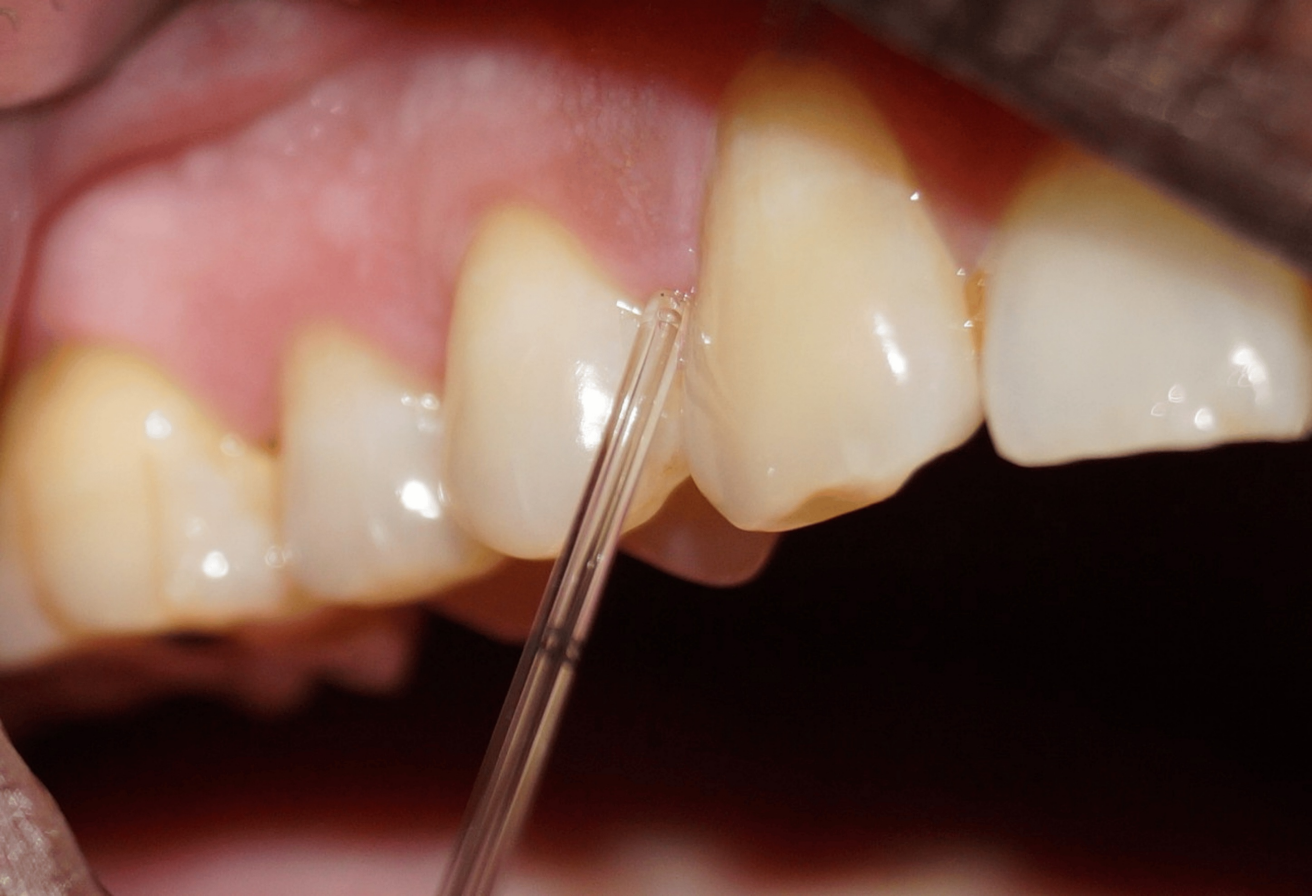
Collection of gingival crevicular fluid (GCF) using micro capillary pipette

GCF samples were collected the day after clinical examination. With the patient seated upright, the selected site was gently air-dried and isolated using cotton rolls. A 5 µL microcapillary pipette (Sigma-Aldrich, St. Louis, MO, USA) was used to collect 3 µL of GCF extra-crevicularly within 10-20 minutes (Figure 3).

Samples contaminated with blood or saliva were excluded. The collected GCF samples were immediately stored at -80°C at Asha Hospital and Research Centre, Bangalore, until biomarker analysis.

**Figure 4:**
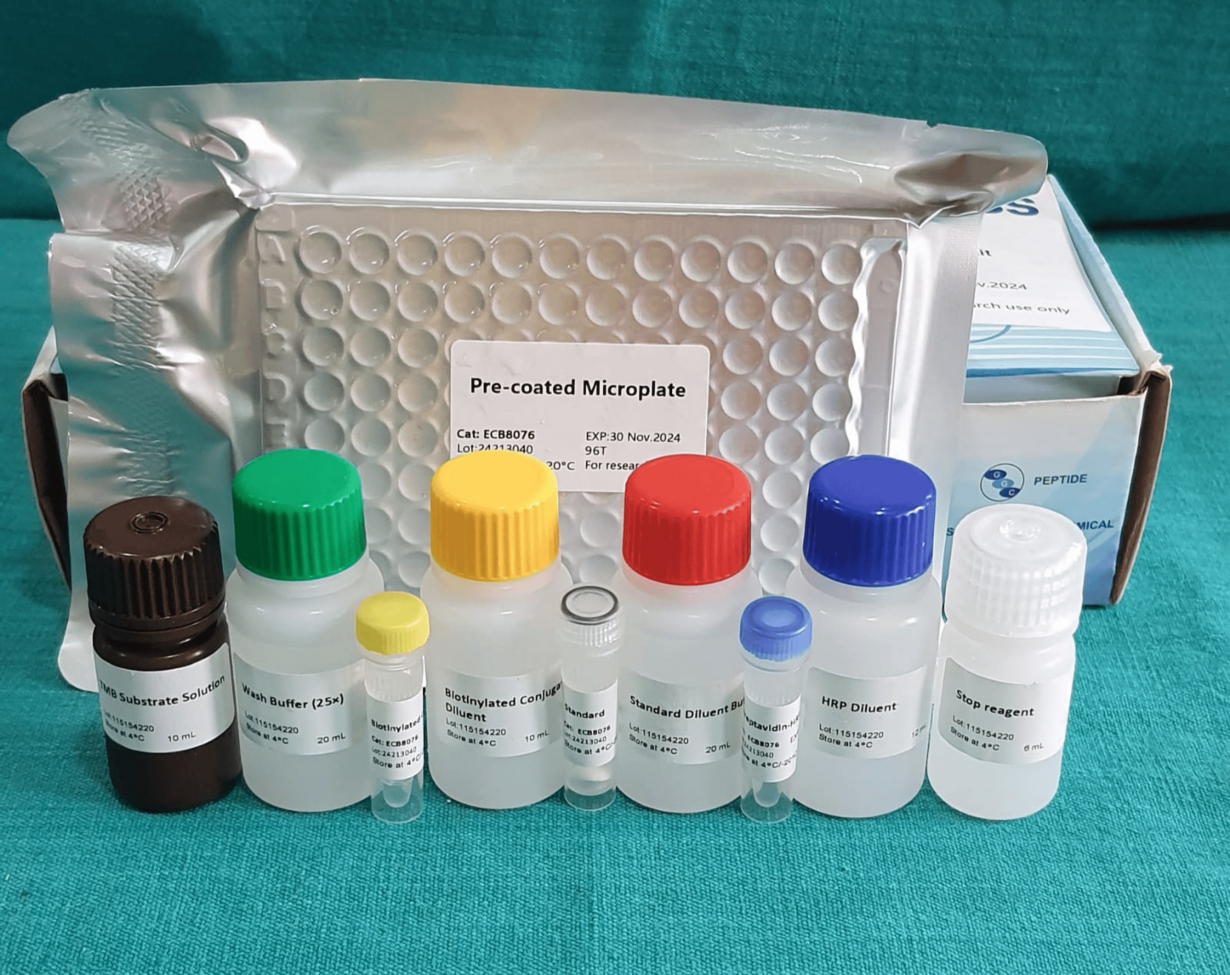
Enzyme-linked immunosorbent assay (ELISA) kit

Estimation of biomarkers was carried out using commercially available sandwich enzyme-linked immunosorbent assay (ELISA) kits (EC Biolabs, New Delhi, India) (Figure 4).

**Figure 5:**
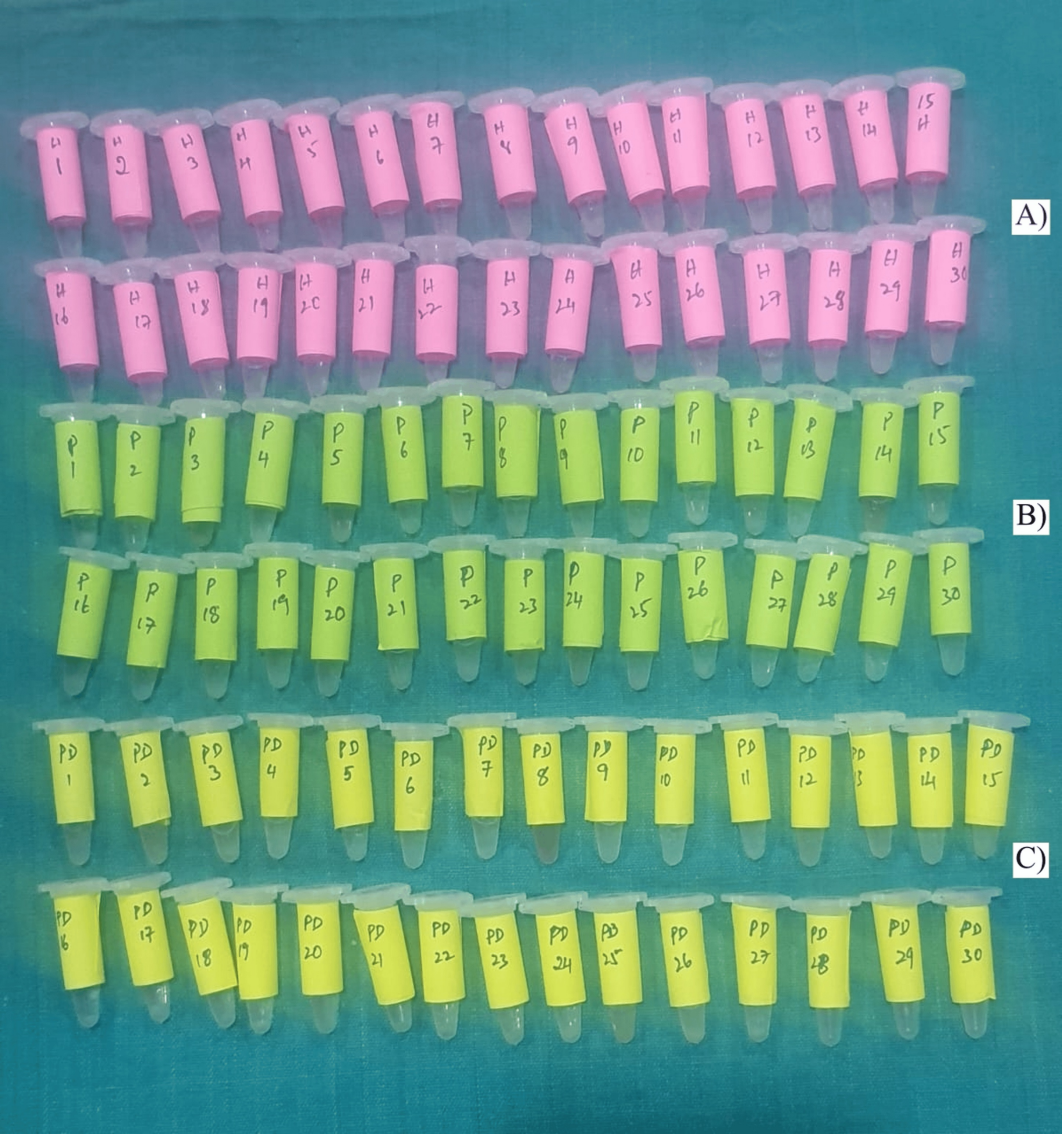
Gingival crevicular fluid (GCF) samples collected and added to Eppendorf tubes containing PBS solution: (A) healthy subjects, (B) subjects with periodontitis, and (C) subjects with periodontitis and type 2 diabetes mellitus (T2DM).

Microtiter plates pre-coated with specific antibodies were incubated with standards and samples, followed by biotin-conjugated antibodies and horseradish peroxidase (HRP)-linked streptavidin. After sequential washing steps (Figure 5), tetramethylbenzidine (TMB) substrate was added, leading to a color change proportional to the biomarker concentration. The absorbance was measured at 450 nm using an ELISA reader, and levels of ghrelin and TNF-α were quantified.

Following baseline GCF collection, all participants received NSPT. The treatment protocol consisted of patient education, motivation, scaling, root planing, and oral hygiene instructions. A structured maintenance program was implemented, including biweekly follow-up visits to reinforce oral hygiene practices over a three-month period. At the end of this phase, GCF samples were recollected from all participants, and periodontal clinical parameters were re-evaluated using the same methodology applied at baseline.

Statistical analysis

The sample size for the present study was calculated using G*Power software (version 3.1.9.7; Heinrich-Heine-Universität Düsseldorf, Düsseldorf, Germany). The estimation was based on a significance level of 5% (α = 0.05), an effect size of 0.34 derived from the findings of Ohta et al. (2011), which reported the mean difference in GCF ghrelin levels between groups, and a statistical power of 80%. The analysis indicated that a minimum of 87 samples would be required. Therefore, the sample size was rounded off to 90, with 30 samples allocated to each of the three study groups.

Statistical analysis was carried out using SPSS for Windows, Version 22.0 (Released 2013; IBM Corp., Armonk, NY, USA). Descriptive statistics were presented as frequencies and proportions for categorical variables, whereas continuous variables were expressed as mean and standard deviation (SD).

The normality of data distribution was assessed using the Shapiro-Wilk test. Results indicated that the data for clinical parameters, body mass measures, and biomarker levels followed a normal distribution. Accordingly, parametric tests were employed for inferential analysis.

For intergroup comparisons, one-way analysis of variance (ANOVA) followed by Tukey’s honestly significant difference (HSD) post hoc test was performed to compare the mean values of clinical parameters, as well as GCF ghrelin and TNF-α levels, both before and after treatment across the three study groups. Within each group, comparisons of pre- and post-treatment values for clinical parameters and biomarker levels were carried out using the paired Student’s t-test.

The relationship between clinical parameters and biomarker levels (GCF ghrelin and TNF-α) before and after treatment in each group was assessed using Pearson’s correlation analysis. To further evaluate predictive associations, stepwise multiple linear regression analysis was employed to determine whether clinical parameters could predict changes in GCF ghrelin and TNF-α levels before and after treatment.

For demographic comparisons, the Kruskal-Wallis test was used to assess differences in mean age among the three groups, while the Chi-square test was applied to compare gender distribution. For all analyses, the level of statistical significance was set at p < 0.05.

## Results

A total of 90 subjects were recruited for the study, comprising 30 individuals in Group I (healthy), 30 individuals in Group II (generalized Stage III Grade B periodontitis without T2DM), and 30 individuals in Group III (generalized Stage III Grade B periodontitis with T2DM). The primary objective was to estimate and correlate ghrelin and TNF-α levels across these three groups.

Sex distribution was comparable among the three groups, with no statistically significant difference observed (p = 0.56). In Group I, 43.3% were males and 56.7% were females. Group II included 56.7% males and 43.3% females, while Group III comprised 53.3% males and 46.7% females as shown in Table 2.

**Table 2:** Gender distribution among the three groups a - Chi Square Test; n - Number of subjects; df - Degree of freedom Group 1: Healthy subjects; Group 2: Periodontitis patients without diabetes; Group 3: Periodontitis patients with diabetes. Chi Square Test Statistics value: 1.156, df = 2, Effect Size (Cramer’s V) = 0.13

Variable	Category	Group 1		Group 2		Group 3		df	p-value
		n	%	n	%	n	%		
Sex	Males	13	43.30%	17	56.70%	16	53.30%	2	0.56^a^
	Females	17	56.70%	13	43.30%	14	46.70%		

The mean age of participants was 44.43 ± 4.30 years in Group I, 46.53 ± 7.75 years in Group II, and 47.53 ± 4.95 years in Group III. The differences in age across the groups were not statistically significant (p = 0.12). The age range of participants was 38-54 years in Group I, 33-60 years in Group II, and 38-60 years in Group III as shown in Table 3.

**Table 3:** Age distribution among the three groups b. Kruskal-Wallis Test Group 1: Healthy subjects; Group 2: Periodontitis patients without diabetes; Group 3: Periodontitis patients with diabetes.

Variable	Category	Group 1		Group 2		Group 3		p-value
		Mean	SD	Mean	SD	Mean	SD	
Age	Mean	44.43	4.3	46.53	7.75	47.53	4.95	0.12^b^
	Range	38 - 54		33 - 60		38 - 60		

Clinical parameters

At baseline, PI values were lowest in Group I (0.95 ± 0.24), while significantly higher scores were observed in Group II (2.18 ± 0.33) and Group III (2.38 ± 0.31) (p < 0.001, Table 4). Similarly, GI was also lowest in Group I (0.47 ± 0.24) compared to Group II (1.64 ± 0.30) and Group III (2.07 ± 0.32), with the differences being highly significant (p < 0.001, Table 4). With respect to BOP, minimal bleeding was recorded in Group I (5.05 ± 2.08), whereas Group II (77.21 ± 4.19) and Group III (77.77 ± 6.70) exhibited markedly higher scores, again with significant intergroup differences (p < 0.001, Table 4). PD was shallowest in Group I (1.84 ± 0.40), while Groups II (5.72 ± 0.45) and Group III (5.97 ± 0.59) showed significantly greater values, confirming the presence of deeper pockets in the periodontitis groups (p < 0.001, Table 4). Finally, at baseline ICAL was comparable in Group II (5.37 ± 0.24 mm) and Group III (5.48 ± 0.32 mm) (p=0.15).

**Table 4:** Comparison of mean values of clinical parameters before treatment * Statistically Significant; p-value (<0.05) a - One-way ANOVA Test; b- Tukey’s post hoc Test PI: Plaque Index; GI: Gingival Index; BOP: Bleeding on probing; PPD: Periodontal probing depth; N: number of participants; SD: standard deviation Group 1: Healthy subjects; Group 2: Periodontitis patients without diabetes; Group 3: Periodontitis patients with diabetes.

Parameters	Groups	N	Mean	SD	p-value ^a^	Sig. Diff	p-value ^b^
PI	Group 1	30	0.95	0.24	<0.001*	1 vs 2	<0.001*
	Group 2	30	2.18	0.33		1 vs 3	<0.001*
	Group 3	30	2.38	0.31		2 vs 3	0.03*
GI	Group 1	30	0.47	0.24	<0.001*	1 vs 2	<0.001*
	Group 2	30	1.64	0.3		1 vs 3	<0.001*
	Group 3	30	2.07	0.32		2 vs 3	0.005*
BOP	Group 1	30	5.05	2.08	<0.001*	1 vs 2	<0.001*
	Group 2	30	77.21	4.19		1 vs 3	<0.001*
	Group 3	30	77.77	6.7		2 vs 3	0.89
PPD	Group 1	30	1.84	0.4	<0.001*	1 vs 2	<0.001*
	Group 2	30	5.72	0.45		1 vs 3	<0.001*
	Group 3	30	5.97	0.59		2 vs 3	0.12

Three months after NSPT, PI values in Group I remained almost unchanged (0.93 ± 0.26, p = 0.20), whereas groups II (1.73 ± 0.28) and III (1.90 ± 0.29) demonstrated statistically significant reductions (p < 0.001, Table 5). For GI, Group I showed a slight, non-significant reduction (0.42 ± 0.19, p = 0.07), while groups II (0.91 ± 0.19) and III (1.52 ± 0.26) exhibited significant improvements (p < 0.001, Table 5). BOP also decreased across all groups, with values of 4.05 ± 2.08 in Group I, 62.22 ± 4.19 in Group II, and 62.78 ± 6.69 in Group III. These reductions were statistically significant overall (p < 0.001), although the difference between groups II and III was not significant (p = 0.89, Table 5). With respect to PD, minimal change was observed in Group I (1.81 ± 0.37), whereas groups II (4.53 ± 0.39) and III (4.71 ± 0.44) showed significant reductions (p < 0.001, Table 5), reflecting greater therapeutic gains in periodontitis patients. Furthermore, CAL demonstrated significant improvement in both groups II and III, with mean gains of 4.99 ± 0.37 mm and 4.96 ± 0.36 mm, respectively (p < 0.001). No significant intergroup difference was noted (p = 0.75), although Group III exhibited a marginally greater improvement. 

**Table 5:** Comparison of mean values of clinical parameters after treatment * Statistically Significant; p-value (<0.05) a - One-way ANOVA Test; b - Tukey’s post hoc Test PI: Plaque Index; GI: Gingival Index; BOP: Bleeding on probing; PPD: Periodontal probing depth; N: number of participants; SD: standard deviation Group 1: Healthy Subjects; Group 2: Periodontitis patients without diabetes; Group 3: Periodontitis patients with diabetes.

Parameters	Groups	N	Mean	SD	p-value ^a^	Sig. Diff	p-value ^b^
PI	Group 1	30	0.93	0.26	<0.001*	1 vs 2	<0.001*
	Group 2	30	1.73	0.28		1 vs 3	<0.001*
	Group 3	30	1.9	0.29		2 vs 3	0.04*
GI	Group 1	30	0.42	0.19	<0.001*	1 vs 2	<0.001*
	Group 2	30	0.91	0.19		1 vs 3	<0.001*
	Group 3	30	1.52	0.26		2 vs 3	<0.001*
BOP	Group 1	30	4.05	2.08	<0.001*	1 vs 2	<0.001*
	Group 2	30	62.22	4.19		1 vs 3	<0.001*
	Group 3	30	62.78	6.69		2 vs 3	0.89
PPD	Group 1	30	1.81	0.37	<0.001*	1 vs 2	<0.001*
	Group 2	30	4.53	0.39		1 vs 3	<0.001*
	Group 3	30	4.71	0.44		2 vs 3	0.2

Biomarkers

At baseline, GCF ghrelin levels were lowest in Group I, while progressively higher values were observed in groups II and III (Table 6). Likewise, TNF-α levels were also lowest in Group I, whereas groups II and III demonstrated markedly elevated values (Table 6). Intergroup comparisons confirmed these differences to be statistically significant (p < 0.001) (Table 6).

**Table 6:** Comparison of mean GCF ghrelin and TNF-α levels (in pg/ml) before treatment between the three groups * Statistically Significant; p-value (<0.05) a - One-way ANOVA Test; b - Tukey’s post hoc Test PI: Plaque Index; GI: Gingival Index; BOP: Bleeding on probing; PPD: Periodontal probing depth; N: number of participants; SD: standard deviation; TNF-α: Tumor Necrosis Factor alpha; GCF: gingival crevicular fluid Group 1: Healthy Subjects; Group 2: Periodontitis patients without diabetes; Group 3: Periodontitis patients with diabetes.

Parameters	Groups	N	Mean	SD	p-value ^a^	Sig. Diff	p-value ^b^
Ghrelin	Group 1	30	3084.24	235.95	<0.001*	1 vs 2	<0.001*
	Group 2	30	3532.26	160.42		1 vs 3	<0.001*
	Group 3	30	3758.85	46.1		2 vs 3	0.001*
TNF-α	Group 1	30	59.88	3.59	<0.001*	1 vs 2	<0.001*
	Group 2	30	78.4	7.56		1 vs 3	<0.001*
	Group 3	30	111.18	12.19		2 vs 3	<0.001*

Following NSPT, ghrelin levels showed a significant reduction across all groups (p < 0.001), with post-treatment values declining to 2438.23 ± 56.26 pg/ml in Group I, and proportionally lower values in groups II and III. The greatest reduction was noted in Group III (−968.60 pg/ml), followed by Group II (−897.06 pg/ml) and Group I (−646.00 pg/ml) (Table 7). Similarly, TNF-α levels decreased significantly after treatment (p < 0.001), with post-treatment values of 44.33 ± 8.42 pg/ml in Group I and substantially reduced levels in groups II and III. The largest decline occurred in Group III (−27.61 pg/ml), followed by Group I (−15.55 pg/ml) and Group II (−9.73 pg/ml), reflecting a marked reduction in inflammatory burden following periodontal therapy (Table 7). 

**Table 7:** Comparison of mean GCF ghrelin and TNF-α levels (in pg/ml) three months after treatment between the three groups * Statistically Significant; p-value (<0.05) a - One-way ANOVA Test; b - Tukey’s post hoc Test PI: Plaque Index; GI: Gingival Index; BOP: Bleeding on probing; PPD: Periodontal probing depth; N: number of participants; SD: standard deviation; TNF-α: Tumor Necrosis Factor alpha; GCF: gingival crevicular fluid Group 1: Healthy Subjects; Group 2: Periodontitis patients without diabetes; Group 3: Periodontitis patients with diabetes.

Parameters	Groups	N	Mean	SD	p-value ^a^	Sig. Diff	p-value ^b^
Ghrelin	Group 1	30	2438.23	56.26	<0.001*	1 vs 2	<0.001*
	Group 2	30	2635.19	61.18		1 vs 3	<0.001*
	Group 3	30	2790.25	25.91		2 vs 3	0.001*
TNF-α	Group 1	30	44.33	8.42	<0.001*	1 vs 2	<0.001*
	Group 2	30	68.67	5.02		1 vs 3	<0.001*
	Group 3	30	83.57	7.04		2 vs 3	<0.001*

Table 8 presents the correlation between clinical parameters and GCF levels of ghrelin and TNF-α before treatment. In Group I, ghrelin showed weak, non-significant correlations with all clinical parameters and HbA1c (r = 0.03-0.13). Similarly, TNF-α demonstrated only weak, non-significant associations with plaque index, gingival index, bleeding on probing, pocket depth, and HbA1c.

**Table 8:** Pearson's correlation test to assess the relationship between clinical parameters and HbA1c levels with GCF ghrelin and TNF-α levels before treatment in each group * Statistically Significant; p-value (<0.05) PI: Plaque Index; GI: Gingival Index; BOP: Bleeding on probing PPD: Periodontal probing depth; N: number of participants; SD: standard deviation; TNF-α: Tumor Necrosis Factor alpha; r: Pearson’s correlation coefficient; GCF: gingival crevicular fluid Group 1: Healthy Subjects; Group 2: Periodontitis patients without diabetes; Group 3: Periodontitis patients with diabetes.

Groups	Markers	values	Ghrelin	PI	GI	BOP	PD	ICAL	HbA1c
Group 1	Ghrelin	r	0.05	0.03	0.09	0.05	0.07	..	0.13
		p-value	0.8	0.86	0.64	0.78	0.73	.	0.51
	TNF-α	r	1	0.26	0.35	0.35	0.33	..	0.13
		p-value	..	0.17	0.06	0.06	0.07	..	0.49
Group 2	Ghrelin	r	-0.26	-0.14	0.05	-0.34	-0.01	-0.08	-0.1
		p-value	0.17	0.45	0.81	0.15	0.96	0.64	0.62
	TNF-α	r	1	0.41	0.82	0.57	0.55	0.49	0.28
		p-value	..	0.02*	<0.001*	0.001*	0.002*	0.006*	0.13
Group 3	Ghrelin	r	-0.31	-0.09	0.11	-0.23	-0.21	-0.19	-0.2
		p-value	0.09	0.64	0.55	0.22	0.27	0.32	0.29
	TNF-α	r	1	0.27	0.63	0.9	0.72	0.81	0.51
		p-value	..	0.15	<0.001*	<0.001*	<0.001*	<0.001*	0.004*

In Group II, ghrelin again displayed weak, negative, and non-significant correlations with the clinical parameters. By contrast, TNF-α revealed strong, statistically significant correlations with plaque index (r = 0.41, p = 0.02), gingival index (r = 0.82, p < 0.001), bleeding on probing (r = 0.57, p = 0.001), pocket depth (r = 0.55, p = 0.002), and initial CAL (r = 0.49, p = 0.006). Its association with HbA1c was weak and not significant (r = 0.28, p = 0.13).

In Group III, ghrelin consistently showed weak, negative, and non-significant correlations with all parameters and HbA1c. Conversely, TNF-α demonstrated strong, statistically significant correlations with gingival index (r = 0.63), bleeding on probing (r = 0.90), pocket depth (r = 0.72), initial CAL (r = 0.81), and HbA1c (r = 0.51, p = 0.004).

Table 9 presents the regression analysis conducted before treatment, predicting GCF ghrelin and TNF-α levels based on selected clinical parameters. In Group II, TNF-α levels were predicted by GI, BOP, and ICAL. Among these, GI had the strongest effect (β = 16.19, p = 0.02), followed by BOP (β = 0.58, p = 0.001) and ICAL (β = 7.21, p = 0.02). The model explained 80% of the variance (R² = 0.80). Pre-treatment, a 1-unit increase in GI, a 1% rise in BOP, and a 1 mm increase in ICAL corresponded to increases of 16.19, 0.58, and 7.21 pg/ml in TNF-α levels, respectively.

**Table 9:** Multivariate stepwise linear regression analysis to predict the GCF ghrelin and TNF-α levels using clinical parameters before treatment in each group DV: Dependent Variable; IV: Independent Variable; β: Beta coefficient / regression coefficient; R²: Coefficient of determination; t: test statistic in regression. BOP: Bleeding on probing; PPD: Periodontal probing depth; ICAL: Interdental clinical attachment loss; TNF-α: Tumor Necrosis Factor alpha; GCF: Gingival Crevicular Fluid Group 1: Healthy Subjects; Group 2: Periodontitis patients without diabetes and Group 3: Periodontitis patients with diabetes.

Groups	DV	IV	Unstd. Coefficients		t	p-value	R^2^
			β	Std. Error			
Group 2	TNF-α	Constant	-31.29	17.17	-1.823	0.08	0.8
		GI	16.19	2.27	7.135	0.02*	
		BOP	0.58	0.16	3.625	0.001*	
		ICAL	7.21	2.82	2.56	0.02*	
Group 3	TNF-α	Constant	-33.04	10.05	-3.288	0.003*	0.87
		GI	7.11	2.76	2.578	0.02*	
		BOP	1.15	0.17	6.74	0.02*	
		ICAL	5.18	1.77	2.936	0.007*	

In Group III, TNF-α levels were likewise predicted by GI (β = 7.11, p = 0.02), BOP (β = 1.15, p = 0.02), and ICAL (β = 5.18, p = 0.007). This model accounted for 87% of the variance (R² = 0.87). Pre-treatment, each 1-unit increase in GI, 1% rise in BOP, and 1 mm increase in ICAL resulted in increases of 7.11, 1.15, and 5.18 pg/ml in TNF-α levels, respectively.

Table 10 presents the correlation between clinical parameters and GCF levels of ghrelin and TNF-α after treatment. In Group I, ghrelin showed weak, non-significant correlations with plaque index (r = -0.04), gingival index (r = -0.16), bleeding on probing (r = -0.23), and pocket depth (r = -0.24), while a weak positive association was observed with initial clinical attachment level (r = 0.19). Similarly, TNF-α demonstrated only weak, non-significant correlations with all parameters, including plaque index (r = 0.15), gingival index (r = 0.25), bleeding on probing (r = 0.38), and pocket depth (r = 0.29).

**Table 10:** Pearson's correlation test to assess the relationship between clinical parameters with GCF ghrelin and TNF-α levels after treatment in each group * Statistically Significant; p-value (<0.05) PI: Plaque Index; GI: Gingival Index; BOP: Bleeding on probing PPD: Periodontal probing depth; N: number of participants; SD: standard deviation; TNF-α: Tumor Necrosis Factor alpha; r: Pearson’s correlation coefficient; GCF: gingival crevicular fluid Group 1: Healthy Subjects; Group 2: Periodontitis patients without diabetes; Group 3: Periodontitis patients with diabetes.

Groups	Markers	values	Ghrelin	PI	GI	BOP	PD	ICAL
Group 1	Ghrelin	r	1	-0.04	-0.16	-0.23	-0.24	0.19
		p-value		0.85	0.41	0.22	0.21	0.314
	TNF-α	r	-0.036	1	0.15	0.25	0.38	0.29
		p-value	0.851		0.42	0.2	0.11	0.18
Group 2	Ghrelin	r	1	-0.23	-0.29	-0.29	-0.6	0.32
		p-value		0.23	0.12	0.12	<0.001*	0.04*
	TNF-α	r	-0.225	1	0.01	0.17	0.07	0.08
		p-value	0.233		0.97	0.38	0.72	0.68
Group 3	Ghrelin	r	1	0.01	0.05	-0.04	-0.05	0.03
		p-value		0.98	0.81	0.84	0.79	0.87
	TNF-α	r	0.005	1	0.19	0.28	0.4	0.44
		p-value	0.98		0.31	0.13	0.03*	0.02*

In Group II, ghrelin exhibited weak, negative correlations with plaque index (r = -0.23), gingival index (r = -0.29), and bleeding on probing (r = -0.29). A moderate, statistically significant negative correlation was found with pocket depth (r = -0.60, p < 0.001). Ghrelin also showed a weak positive correlation with initial CAL (r = 0.32, p = 0.04), though not statistically significant. TNF-α displayed only weak, non-significant correlations across all parameters, including plaque index (r = 0.01), gingival index (r = 0.17), bleeding on probing (r = 0.07), pocket depth (r = 0.08).

In Group III, ghrelin demonstrated negligible, non-significant correlations with clinical parameters (ranging from -0.05 to 0.12). By contrast, TNF-α exhibited moderate, statistically significant positive correlations with pocket depth (r = 0.40, p = 0.03) and initial CAL (r = 0.44, p = 0.02). Other correlations, such as gingival index (r = 0.19), bleeding on probing (r = 0.28), did not reach statistical significance.

Table 11 presents the post-treatment regression analysis predicting GCF ghrelin and TNF-α levels based on selected clinical parameters. In Group II, GCF ghrelin levels were significantly predicted by BOP and PD. The constant term (2941.95 ± 151.74, p < 0.001) was significant. BOP showed a negative effect (β = -9.24 ± 2.00, p < 0.001), while PD exhibited a positive effect (β = 59.08 ± 21.62, p = 0.01). This indicates that a 1% increase in BOP was associated with a decrease of 9.24 pg/ml in ghrelin, whereas a 1 mm increase in PD corresponded to an increase of 59.08 pg/ml. Overall, the model explained 46% of the variance (R² = 0.46).

**Table 11:** Multivariate stepwise linear regression analysis to predict the GCF ghrelin and TNF-α levels using clinical parameters after treatment in each group * - Statistically Significant; p-value (<0.05) DV: Dependent Variable; IV: Independent Variable; β: Beta coefficient / regression coefficient; R²: Coefficient of determination; t: test statistic in regression. BOP: Bleeding on probing; PPD: Periodontal probing depth; ICAL: Interdental clinical attachment loss; TNF-α: Tumor Necrosis Factor alpha; GCF: Gingival Crevicular Fluid Group 1: Healthy Subjects; Group 2: Periodontitis patients without diabetes; Group 3: Periodontitis patients with diabetes.

Groups	DV	IV	Unstd. Coefficients		t	p-value	R^2^
			β	Std. Error			
Group 2	GCF Ghrelin	Constant	2941.95	151.74	19.388	<0.001*	0.46
		BOP	-9.24	2	-4.62	<0.001*	
		PD	59.08	21.62	2.732	0.01*	
Group 3	GCF TNF-α	Constant	50.47	12.79	3.947	<0.001*	0.17
		ICAL	7.02	2.7	2.599	0.02*	

In Group III, TNF-α levels were significantly predicted by ICAL. Both the constant (50.47 ± 12.79, p < 0.001) and the ICAL coefficient (β = 7.02 ± 2.70, p = 0.02) were significant, indicating a positive association. Specifically, a 1 mm gain in ICAL was associated with an increase of 7.02 pg/ml in TNF-α. The model accounted for 17% of the variance (R² = 0.17).

## Discussion

This study explored the relationship between ghrelin, TNF-α levels, and periodontal parameters in individuals with periodontitis, with and without T2DM. The investigation was inspired by evidence suggesting that ghrelin can inhibit LPS-induced TNF-α production in oral epithelial cells [14],highlighting its potential immunomodulatory role.

The age ranges across Group I (38-54 years), Group II (33-60 years), and Group III (38-60 years) were comparable (means: 44.43, 46.53, and 47.53 years, respectively), reflecting an intentional standardization despite the known age-related rise in periodontitis and T2DM prevalence. Gender distribution showed a higher proportion of males in the periodontitis groups, likely due to poorer oral hygiene practices. This result is coherent with findings by Shiau et al. (2010) [15] and Eke et al. (2015) [16], who reported higher periodontitis prevalence in males. Additionally, female sex hormones may offer protective effects by enhancing humoral immunity and reducing proinflammatory responses as stated by Ioannidou E (2017) [17].

In the current study, baseline PI and GI scores were markedly higher in the diseased compared to the healthy group, with mean PI values of 2.18 ± 0.33 and 2.38 ± 0.31, and GI scores of 1.64 ± 0.30 and 2.07 ± 0.32, in groups II and III respectively. The positive correlation between PI and GI underscores the role of dental plaque in initiating gingival inflammation, as supported by Ramirez et al. (2020) and Ayan et al. (2023) [18,19]. Despite similar PI scores between groups II and III, Group III exhibited considerably higher GI scores, possibly caused by the upregulated inflammatory response associated with T2DM. This is supported by Karthik et al. (2018) and Zhao et al. (2023), who reported that hyperglycaemia-induced AGEs activate pro-inflammatory pathways and cytokine release in the periodontium [20,21].

After three months of NSPT, PI and GI scores significantly reduced in all groups, with Group III still showing higher reduction in GI compared to other groups. This reduction reflects improved periodontal health due to plaque removal and decreased inflammation. Our findings are in line with studies by Gomathi et al. and Amith et al., which emphasized the effectiveness of NSPT in reducing plaque, gingival inflammation, and clinical symptoms, especially in T2DM patients [22,23].

Baseline PD values in the present study were 1.84 ± 0.40 for Group I, 5.72 ± 0.45 for Group II, and 5.97 ± 0.59 for Group III. The absence of a markedly significant difference in PD between groups II and III may be attributed to similar plaque accumulation and the enrolment of patients with well-controlled type 2 diabetes mellitus. These findings echo with those of Kowall et al., who reported no significant association between mean CAL, PPD, or edentulism in patients with well-controlled T2DM (HbA1c <7%) [24]. Also, Stoicescu M et al. observed that subjects presenting with poorly controlled diabetes (HbA1c ≥7%) exhibited a higher prevalence of periodontal pockets ≥5 mm and experienced greater attachment loss compared to those with better glycemic control [25].

In our study, a statistically significant reduction in probing depth was observed within all groups from baseline to three months following NSPT. This improvement is likely due to reduced inflammation after plaque removal, promoting soft tissue healing and pocket shrinkage. Proye et al. reported that even a single session of NSPT can significantly reduce pocket depth, attributing this to inflammation resolution and gingival tissue reattachment [26]. Supporting this, Jiao et al., in a large-scale study involving 10,789 chronic periodontitis patients, confirmed the effectiveness of NSPT in managing periodontal disease. Their study also further highlighted that treatment outcomes are largely influenced by individual baseline characteristics, highlighting the importance of personalized periodontal care [27].

Intergroup comparisons revealed a similar mean PD reduction of ~1 mm in both Group II and Group III, possibly linked to similar baseline PD and glycaemic control. Our results are in line with those of Kolte et al., who reported a 1.4 mm reduction in probing depth three months after NSPT in well-controlled type 2 diabetes mellitus patients, as compared to supragingival scaling. This improvement was attributed to the effectiveness of subgingival debridement in reducing microbial load and halting disease progression. The study also noted that periodontal destruction in diabetic individuals tends to be more severe, even with minimal plaque, due to compromised host immune response [28]. Similarly, Kocher et al. demonstrated that NSPT resulted in a mean PPD reduction of over 1 mm in well-controlled T2DM patients, comparable to non-diabetic individuals. The reduction was more pronounced at sites with deeper baseline pockets, emphasizing the role of initial disease severity in determining therapeutic outcomes [29].

Ghrelin is a peptide hormone predominantly produced by the stomach, with lesser contributions from the small intestine, pancreas, and brain. Known as the "hunger hormone," it stimulates appetite, promotes fat storage, and supports physiological processes like digestion, inflammation regulation, sleep, and neurological function [30]. In this study, baseline GCF ghrelin levels were 3084.24 pg/ml in Group I (Healthy), 3532.26 pg/ml in Group II (Periodontitis), and 3758.85 pg/ml in Group III (Periodontitis with T2DM), indicating significantly elevated levels in diseased states.

The presence of ghrelin in healthy (Group I) individuals might imply its influence in homeostatic tissue regulation. Ohta et al. proposed that oral epithelial cells and gingival fibroblasts express ghrelin and its receptor (GHS-R), explaining its presence in GCF. The significantly elevated ghrelin levels in periodontitis groups (Group II and Group III), as compared to the healthy group, may reflect a compensatory anti-inflammatory response to periodontal inflammation. Ghrelin has been shown to upregulate the production of anti-inflammatory cytokines such as IL-10 while concurrently downregulating pro-inflammatory mediators including IL-1β and TNF-α, as demonstrated in studies by Waseem et al. [8].

Furthermore, Yılmaz et al. reported significant upregulation in plasma levels of both total and acylated ghrelin among individuals diagnosed with chronic periodontitis among male subjects; however, no measurable correlation was observed between ghrelin levels and clinical periodontal parameters, indicating a complex and possibly multifactorial relationship between ghrelin and periodontal disease pathogenesis [31].

Group III exhibited the highest ghrelin levels, likely due to the combined inflammatory impact of periodontitis and T2DM. Hyperglycemia in T2DM promotes systemic inflammation through the formation of advanced glycation end products (AGEs), which activate NF-κB pathways and trigger cytokine release, worsening periodontal destruction. Similar findings were reported by Mohamed et al. [32], who observed elevated glucoregulatory biomarkers in the GCF of T2DM patients with periodontitis.

It was noted by Mathur et al. that immune cells can produce ghrelin during inflammation, which binds to GHS-R to suppress pro-inflammatory cytokines, highlighting its immunomodulatory role [33]. Furthermore, Barazzoni et al. observed a reverse relationship between insulin levels and total ghrelin concentrations. Deacylated ghrelin, in particular, has been demonstrated to enhance insulin sensitivity and promote glucose uptake via activation of intracellular pathways such as the AKT-glycogen synthase kinase (GSK) signaling axis. In insulin-resistant conditions, such as T2DM, this counteractive mechanism may have contributed to the elevated ghrelin levels observed in Group III of the current study [34].

In the current study, all three groups showed a statistically significant decline in GCF ghrelin levels three months after NSPT, likely reflecting decreased periodontal inflammation post-treatment. This aligns with the findings of Otero et al., who reported reduced ghrelin levels following inflammation resolution in chronic conditions such as rheumatoid arthritis, possibly due to a feedback mechanism where diminished cytokine activity reduces the stimulus for ghrelin secretion [35].

The only study to date assessing ghrelin levels following NSPT was conducted by Devika et al., who evaluated salivary ghrelin levels in patients presenting with chronic periodontitis and reported an increase in the levels of ghrelin after NSPT [36]. The variation between their findings and those of this study might be due to differences in type of sample collected, as ghrelin levels in GCF are reported to be up to 500-fold higher than in saliva [14]. Additionally, the shorter post-treatment assessment interval in their study compared to the present one may have influenced the results. Lyra et al. noted that ghrelin levels typically decrease during the acute phase of wound healing and gradually begin to recover as healing progresses, which could partly account for the variation in findings [37].

TNF-α is a key proinflammatory cytokine primarily secreted by activated macrophages, lymphocytes, and natural killer cells. It plays essential roles in inflammation, cell survival, apoptosis, and tissue homeostasis [38]. In this study, baseline mean TNF-α levels were 59.88 pg/ml in Group I (Healthy), 78.40 pg/ml in Group II (Periodontitis), and 111.18 pg/ml in Group III (Periodontitis with T2DM). The existence of TNF-α in healthy individuals is presumably linked to its role in maintaining epithelial integrity and immune regulation, as described by Osta et al. and Ruder et al. [39,40].

Our results demonstrated a pronounced increase in TNF-α levels in the diseased group compared to the healthy group, consistent with its role in the host immune response to periodontal pathogens like *Aggregatibacter actinomycetemcomitans* and *Porphyromonas gingivalis *[41]. These bacteria release PAMPs such as LPS, which are recognized by PRRs on immune cells, triggering TNF-α production [41]. Group III exhibited the highest TNF-α levels, reflecting a compounded inflammatory effect of periodontitis and T2DM. Singh et al. reported similar findings, attributing elevated TNF-α levels in diabetic patients to AGE-RAGE interactions that activate proinflammatory signalling pathways [42].

Following NSPT, TNF-α levels showed significant reduction across all three groups, indicating reduced inflammation and improved periodontal health. This finding is in line with the results reported by Kolte et al., who attributed the reduction in pro-inflammatory mediators, including TNF-α, to the removal of plaque, calculus, and periodontal pathogens through NSPT [28]. Similarly, Dag et al. also observed significant reductions in TNF-α levels post-NSPT, along with improvements in glycaemic control among diabetic patients. Additionally, the study also reported a reduction in HbA1c levels among well-controlled type 2 diabetes mellitus patients [43].

The healthy group showed modest reductions in ghrelin and TNF-α levels in GCF after NSPT, likely due to the resolution of subclinical inflammation. As per the 2017 periodontal classification, clinical periodontal health allows for minimal inflammation and some plaque presence, suggesting that NSPT can further improve inflammatory status even in clinically healthy individuals [44].

When comparing biomarker levels at baseline, a strong positive correlation was observed between GCF concentrations of ghrelin and TNF-α across all groups, with both markers increasing in tandem with inflammation. Nokhbehsaim et al. reported that while ghrelin initially exerts anti-inflammatory effects by suppressing pro-inflammatory pathways, prolonged bacterial exposure leads to downregulation of GHS-R1a receptors. This reduction in receptor expression diminishes ghrelin’s anti-inflammatory action, contributing to elevated TNF-α production and subsequent periodontal tissue destruction [45].

Post-NSPT, Group III showed a greater reduction in TNF-α levels compared to Group II, likely due to its higher baseline inflammatory burden. By the end of the three-month follow-up period, TNF-α levels in both groups became comparable. The evidence obtained from this research indicates that NSPT can reduce periodontal inflammation locally and may also aid in regulating systemic inflammation in individuals with T2DM.

Limitations

This study has several limitations that must be acknowledged. First, the follow-up period was restricted to three months, which may not be sufficient to evaluate the long-term effects of non-surgical periodontal therapy on inflammatory biomarkers. Second, HbA1c was recorded only at baseline but not after treatment, preventing evaluation of whether NSPT had any beneficial impact on glycaemic control.

## Conclusions

This study highlights the significant interplay between inflammatory biomarkers and periodontal health in subjects with and without T2DM. Elevated levels of GCF ghrelin and TNF-α were observed in periodontitis patients, with the highest levels seen in those with coexisting T2DM, suggesting a synergistic effect of systemic and local inflammation. The observed positive correlation between these biomarkers and clinical parameters at baseline underscores their role in periodontal disease progression.

Notably, three months post-NSPT, there was a marked reduction in clinical parameters and a corresponding negative correlation with GCF ghrelin and TNF-α levels across all groups, demonstrating the effectiveness of NSPT in mitigating periodontal inflammation also inclusive of diabetic status. These findings emphasize the biological role of ghrelin and TNF-α as potential biomarkers for periodontal disease severity and therapeutic response, especially among individuals with systemic comorbidities such as T2DM.
